# Risk of tuberculosis transmission by healthcare workers to children – a comprehensive review

**DOI:** 10.3205/dgkh000452

**Published:** 2023-10-20

**Authors:** Roland Diel, Albert Nienhaus

**Affiliations:** 1 Institute for Epidemiology, University Medical Hospital Schleswig-Holstein, Kiel, Germany; 2 LungClinic Großhansdorf, Airway Research Center North (ARCN), German Center for Lung Research (DZL), Großhansdorf, Germany; 3 Institute for Health Service Research in Dermatology and Nursing (IVDP), University Medical Center Hamburg-Eppendorf, Hamburg, Germany; 4 Institution for Statutory Accident Insurance and Prevention in the Health and Welfare Services (BGW), Hamburg, Germany

**Keywords:** tuberculosis, children, healthcare workers, transmission, contact investigation

## Abstract

**Background:**

Children <15 years are at elevated risk of becoming infected with *M. tuberculosis co*mplex (Mtbc).

**Objective:**

To assess the magnitude of Mtbc transmission by healthcare workers (HCW) to children

**Methods:**

Medline, Google Scholar and Cochrane library were searched to select primary studies in which HCW was the presumed index case and exposed infants and children aged below 15 years were screened for latent TB infection (LTBI).

**Results:**

Of 4,702 abstracts, 19 original case reports covering one HCW each as presumed source case of Mtbc transmission to children, were identified. In sum, 11,511 children, of those 5,881 infants (51.1%), mostly in newborn nurseries, were considered contact persons and underwent tuberculin skin (TST) or Interferon gamma release assay (IGRA) testing. Test positivity was reported in 492/11,511 children (4.3%) coming from 14 studies. When test results considered falsely positive were excluded, the number of latently infected children decreased to 365/10,171 (3.6%). In all studies, the presumed duration of infectivity of the source case was, but the actual intensity and duration of exposure were not, decisive for the initiation of contact investigations. In only two of the studies, the contact time of the children towards the corresponding source case was estimated.

**Conclusions:**

The results of our review suggest that the risk of Mtbc transmission from HCW to children in healthcare setting is considerably lower than reported in household settings. However, as the preselection of pediatric contacts appeared in most cases to be vague, the data found in the literature probably underestimates the actual risk

## Zusammenfassung

**Hintergrund:**

Kinder unter 15 Jahren haben ein erhöhtes Risiko, sich mit *M. tuberculosis complex* (Mtbc) zu infizieren.

**Zielsetzung:**

Bewertung des Ausmaßes der Mtbc-Übertragung durch medizinisches Personal (MP) auf Kinder.

**Methoden:**

Medline, Google Scholar und die Cochrane-Bibliothek wurden durchsucht, um Originalstudien zu finden, in denen MP der mutmaßliche Indexfall war, und exponierte Säuglinge und Kinder unter 15 Jahren auf eine latente TB-Infektion (LTBI) untersucht wurden.

**Ergebnisse:**

Von 4.702 Abstracts wurden 19 Fallberichte identifiziert, die jeweils einen Angehörigen des MP als mutmaßliche Quelle einer Mtbc-Übertragung auf Kinder dokumentierten. Insgesamt wurden 11.511 Kinder, davon 5.881 Säuglinge (51,1%), meist in Neugeborenen-Stationen, als Kontaktpersonen betrachtet und einem Tuberkulin-Hauttest (TST) oder einem Interferon-Gamma-Release-Assay (IGRA) unterzogen. Ein positiver Test wurde nur bei 492/11.511 Kindern (4,3%) aus 14 Studien gefunden. Schließt man die als falsch positiv eingestuften Testergebnisse aus, sinkt die Zahl der latent infizierten Kinder weiter auf 365/10.171 (3,6%). In allen Studien war die vermutete Dauer der Infektiosität des Ausgangsfalls, nicht aber die tatsächliche Intensität und Dauer der Exposition, entscheidend für die Einleitung von Umgebungs-untersuchungen. Nur in zwei der Studien wurde die Kontaktzeit der Kinder gegenüber dem entsprechenden Ausgangsfall geschätzt.

**Schlussfolgerungen:**

Die Ergebnisse unserer Überprüfung deuten darauf hin, dass das Risiko einer Mtbc-Übertragung durch medizinisches Personal auf Kinder deutlich geringer ist als in Haushalten. Da die Vorauswahl der Kinderkontakte jedoch meist vage war, wird das tatsächliche Risiko wahrscheinlich unterschätzt.

## Introduction

Tuberculosis (TB) is considered an important situational hazard for children, especially for those who are presented for medical attention in hospitals and medical offices. This can be due to the presence in such settings of yet-undiagnosed or untreated TB cases, and/or to inadequate infection control measures. It has been estimated that paediatric cases account for 11% of the global TB cases [1] and that most of them occur in infants and children under the age of 5 years [2]. Of the global Tb deaths among HIV-negative people, 54% were in men, 32% were in women and 14% were in children aged <15 years [1]. 

Exposure of children to TB-afflicted HCW in paediatric clinics and paediatricians’ offices has, to date, been insufficiently investigated. Moreover, it has been systematically examined only in Schepisi’s 2015 review [3]. For this reason, we conducted an update of reports on Mtbc transmission to children in healthcare settings, now with a particular emphasis on the procedures of contact preselection, i.e., infection control practices, in the facilities examined.

## Methods

### Study selection

We performed a thorough literature review based on the PubMed electronic bibliographic database, the Cochrane library and Google Scholar. The following terms were used in Boolean searching: “paediatric,” “children,” “HCW”, “healthcare workers,” “transmission” and “tuberculosis”. Relevant studies were independently selected by two reviewing authors (RD and AN), who screened each article title and abstract initially, and then went on to review an article’s full text as required. 

Any studies published in peer-reviewed journals that provided original data on suggested nosocomial Mtbc transmission to children aged below 15 years by health care workers (HCWs) were considered, without restriction to publication date. Excluded were reviews, guidelines, articles in languages other than English or French, and articles with a central theme diverging from or not related to nosocomial transmission of TB to children. No restrictions were made regarding study design, patient subpopulation, or data collection (prospective or retrospective).

### Data extraction

The following data were extracted from the selected publications: type of healthcare setting, number of exposed infants (from birth to 1 year old) and elder children aged below 15 years, proportion of children in the exposed group with active TB at the time of screening, proportion with latent TB, testing method, possible time of exposure towards the presumed source case, presence of ventilation systems, if any, proportion of BCG-vaccinated children among the test-positives, contact time in hours. Infants and elder children were listed separately. Cases of infants or children who had presented with tuberculosis disease, i.e., whose cases called for the subject investigation, were not included in our compilation.

## Results

Out of 741 (Medline), 3,950 (Google Scholar), and 16 (Cochran Library) abstracts, 20 publications including a total of 19 HCW as presumed source cases were found. One study was excluded: it reported simply that, of the children believed to have been exposed to an HCW with TB, no child had developed active TB. No LTBI screening, however, had been performed [4]. The other 19 studies were analyzed in-depth to compose the present article [5], [6], [7], [8], [9], [10], [11], [12], [13], [14], [15], [16], [17], [18], [19], [20], [21], [22], [23]. 

The studies came from seven countries and covered a publication period from 1991 to 2021. Most were from the USA (5/19, or 26%), France (3, or 16%), the UK (2), Canada (2), and Italy (each 2, or 11%). Further source countries were Australia (1), The Netherlands (1), Japan (1) and Korea (1) and Singapore (1) (see Table 1 (Tab. 1)). 

Of the 19 studies, 12 reported the exposure of HCWs in neonatal intensive units (NICUs) by premature infants born before the 37^th^ week of pregnancy. One study [8] addressed a micro-epidemic of pulmonary TB transmission in two community dental clinics and one study [20] the occasional contact of young children attending a vaccination clinic to a TB-afflicted HCW. 

In sum, 11,511 children, of those 5,881 infants (51.1%), mostly in new-born nurseries, were considered contact persons and underwent tuberculin skin (TST) or Interferon gamma release assay (IGRA) testing. In total, 24 infants or elder children were reported to have active tuberculosis disease. With exception of the 15 children who developed active TB after direct Mtbc transmission by a dentist during tooth extraction, most of the juvenile cases served to trigger the search for the index person and the subsequent contact investigations. Their identification was hence not attributable to the contact investigation itself. Test positivity was reported in only 492/11,511 children (4.3%) and in only 14 of the 19 studies. In the other five studies [7],[12], [14], [17], [20], no positive test results were reported. In all 19 studies, LTBI screening was performed by different types if tuberculin skin testing. In most cases the Mantoux method was applied, but two studies [6, 8] relied on the Heaf test and one study [7], in part, used the tine-test. 

The study with the highest test-positivity was Oh’s Korean study [23] where 314 infants were investigated in which 134 (42.7%) had a positive TST. A primary conclusion was that the children had freshly acquired latent infection due to extensive pulmonary Mtbc transmission by a nurse. However, 91.7% of the subject infants had been BCG-vaccinated. As TST reactions in BCG-vaccinated children had been interpreted using the same criteria as for those who were not BCG-vaccinated, and the TST results in non-BCG-vaccinated were not provided by the authors, it can be assumed that a considerable number of TST results classified as LTBI positive may have simply been due to vaccine-induced cross reactions. 

In three studies [16], [18], [22] IGRA and Mantoux testing were performed simultaneously. In two of these studies, two [16] and eight [22] TST-positive contacts were BCG-vaccinated, but IGRA-negative, and therefore it could be assumed that no LTBI was present. Vice versa, against all probability, 118 of the 1,340 infants (8.8%) in Borgia’s report [18] were tested IGRA-positive but TST-negative, leading to suspicion that the IGRA results may have been falsely positive. When excluding the results of these three studies, the number of latently infected children further decreases to 365/10,171 (3.6%). 

Based on mention, we assume that a face mask was consistently worn only by the index case in Ohno’s study [16] and occasionally in [22]. The infectious period of the single index as a basis for the contact investigations varied between 16 days [14] and 12 months [21]. In all of the studies, children who were potentially exposed during these infectious periods were selected as contact persons without any preselection according to the actual contact time or degree infectivity of the index person; only in two studies was the presumed contact time of the children towards their corresponding index person estimated, and that retrospectively, with on average 30 [17] and 10–20 [21] minutes, respectively. Of note, no confirmation of tuberculosis disease of the presumed index cases by culture was available in six of the 19 studies [5], [6], [8], [12], [21], [22]. Only three studies [7], [16], [20] reported the presence of a ventilation system. 

## Discussion

Despite an initially large number of citations on the topic, we located only very few original studies that addressed nosocomial transmission from HCW to children. More than half of the 19 studies we found were from the field of neonatology. Additionally, questions of methodology, in several cases, challenge the meaningfulness of the results 

First of all, in six presumed index cases, culture confirmation of the tuberculosis disease was missing, making it difficult to draw clear conclusions about the infectiousness of the index persons. In Steiner’s study [4] it is emphasized that the nursery’s aide, the presumptive source case, although she never showed any symptoms and was negative both in sputum smear and in the culture, could have at times been infectious. The study provides no information concerning further diagnostic measures for the detection of Mtbc, e.g., bronchoscopy. Results of the therapeutic outcome (improvement of the x-ray findings?) under combined antituberculosis therapy are missing. Strictly speaking, it remains unclear whether this was a case of infectious pulmonary TB at all. Thus, is not surprising that none of the 1,647 tested children was TST positive. Seen differently, it seems extremely unlikely that the two new-borns suffering from tuberculosis and found to be infected in the nursery before the beginning of the contact investigation could be caused by this index person. Genotyping, which was not available at the time, might have been able to provide key evidence in this case. 

Furthermore, a positive TST is on its own poor evidence of the presence of LTBI. As prior BCG-vaccination is reported in all the studies, reliance on the TST as a measure of transmission is most problematic. Unfortunately, when IGRAS are not available for contact testing of BCG-vaccinated children, distinguishing a true-positive TST reaction (one due to infection) from a vaccine-induced reaction is impossible [24]. The lack of discrimination between BCG-induced TST positivity and positivity due to true infection can, for example, be seen in Ohno’s study [16] where the two TST-positive infants who were also BCG-vaccinated tested negative with the Quantiferon-TB Gold In-Tube test. Therefore, the “true rate” of converters representing fresh infections in those studies that included BCG-vaccinated children is probably lower than described there. 

Even if one accepts the study results as presented in the individual works, irrespective of the methodological problems described above, the average percentage of positive test results indicating LTBI remains very low at 4.3%. Of note, the published literature tells a quite different story. For example, in Fox’s [25] meta-analysis, children aged below 5 years, when tested as household contacts, were found to be infected, on average, in 16.3% of cases [95% CI 9.2–27.0]. Singh et al. [26] even reported a mean LTBI prevalence in 33.8% in children who, in their household, came into contact with adults afflicted with pulmonary TB disease. Sixty-five of these (68.4%) were contacts of sputum-positive adults, and 30 (31.6%), surprisingly, were contacts of the sputum-negative. 

Reflection is required as to what may behind the very low incidence of infection in the studies we found. What stands out is that the periods over which exposure to the index cases might have occurred are, in our 19 studies, quite long. They range from 16 days to 12 months and leave open the question as to whether many of the children participating in the investigation were exposed at all. The low percentage of positive LTBI tests cannot be attributed to accompanying protective measures as in only one study the index person had worn a face mask continuously. Furthermore, an active ventilation system (exchanging ambient air at least 6–7 times/h) that might have reduced the risk of transmission by aerosols such as MTB was only reported in three studies [7], [16], [20]. 

More important for estimating the true risk of becoming infected would be the actual cumulative time of exposure of the suspected contacts to the index cases, about which only little information is available: Only two studies address the cumulative number of hours of explosion: In Bagdasarian’s study [22] only 158 of 464 persons aged below 18 years were screened and only 12 of the total of all exposed children and adolescents had an exposure time of at least 40 hrs. Despite clearly documented contact with the index case, all children who tested Mantoux positive were BCG-vaccinated and IGRA negative, and therefore cannot considered freshly infected. Luzatti et al. [21], in their letter on latent infection of children <6 years, state that even an occasional contact <8 hr may be sufficient for Mtbc transmission. In the same report, the group found an infection rate of only 0.2% among children having each spent 10–20 minutes in close contact with, i.e. receiving vaccination from, a highly contagious physician. Although this contact time was appreciable, it was apparently not long enough – or the exposure was not intense enough – to infect a larger number of children. 

Therefore, careful collection of actual contact times rather than theoretical exposure periods towards an HCW with infectious pulmonary TB could make contact investigations more effective and reduce unnecessary testing. The 2023 update of the German recommendations on contact tracing [26] contain a rule to require 8-hours cumulative exposure time as a prerequisite for environmental testing, even in children, if there was no evidence of direct “face-to-face” contact. Importantly, in cases of doubt, especially where the index case had a history of coughing, an immediate test with IGRA or THT as well as an x-ray examination of the lungs is always required, irrespective of the cumulative exposure time.

## Conclusions

The results of our comprehensive review suggest that the risk of Mtbc transmission from HCW to children is generally low but may be underestimated. When the preselection of contacts is conducted in a vague manner, as in the studies we considered, the proportion of those truly exposed and testing positive may be expected to be low. Therefore, particular attention should be paid not only to the duration of the contact period but to the intensity of contact as well. In this way, a realistic assessment of actual exposure duration can be made. In this context, asking for face-to-face episodes of contact that includes MTB-spreading behaviours, especially coughing and sneezing, will be more helpful than simply calculating theoretical time of exposure, as was done in all of the studies we found. 

## Notes

### Competing interests

The authors declare that they have no competing interests.

Roland Diel received honoraria and/or travel support for presentations at events sponsored by Hain Lifescience, Mikrogen, Oxford Immunotec, or Qiagen.

The honoraria he received had no influence on the subject matter of this article.

### Authors’ ORCID

- Roland Diel: 0000-0001-8304-7709
- Albert Nienhaus: 0000-0003-1881-7302

## Figures and Tables

**Table 1:**
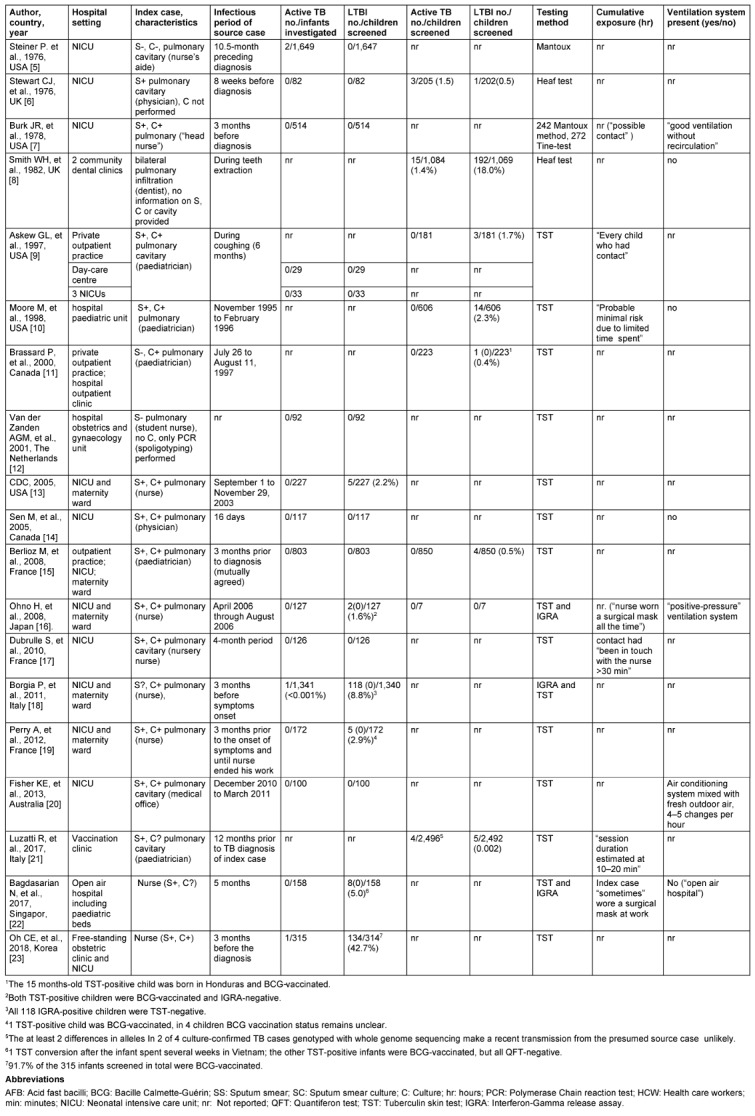
Case reports on children exposed to HCW with TB disease
